# Does Alcohol Afford Nutrition to the Animal Tissues?

**Published:** 1876-05

**Authors:** J. P. Stevens

**Affiliations:** Lee County


ATLANTA

MEDICAL AND SURGICAL JOURNAL

VOL. XIV.] MAY—1876. [No. 2

### Original Communications.

### DOES ALCOHOL AFFORD NUTRITION TO THE ANIMAL TISSUES?

BY J. P. STEVENS, M.D., OF LEE COUNTY.

*Read before the South Georgia Medical Association.*

Inorganic matter, under the influence of solar heat, electricity, friction, or chemical action, rises to a degree of heat commensurate with the intensity and duration of the exciting cause. On the withdrawal of the latter, the object acted on, by conduction or radiation, parts with its heat, and resumes the temperature of the surrounding medium. Man and the mammalia generally, however, possess the inherent property of generating and maintaining the heat of their bodies at a fixed temperature, with slight variations without reference to the extremes of atmospheric heat or cold. The temperature of the human body varies from 98 to 100 degrees; that of birds varies from 100 to 110 degrees; that of reptiles and fishes varies very little from that of the water they inhabit. The Laplander surrounded by perpetual snow and ice, and the rude savage in equatorial regions, maintain the same degree of temperature, under precisely opposite conditions of the atmosphere. Should the temperature of the body at any time fall five or six degrees, below ninety-eight, death is the inevitable consequence. It would be interesting then to enquire, what is the source of animal heat? Ordinary combustion consists in the chemical union of the oxygen of the air with the substance to be ignited. Burn a piece of iron wire in pure oxygen gas, and its consumption is attended with the most brilliant scintillations. The combustion of wood or charcoal arises from the union of oxygen with its carbon, resulting in the constant evolution of carbonic acid gas.

The great German chemist, Leibig, declared that the "mutual action between the elements of the food and the oxygen conveyed by the circulation of the blood to every part of the body, is the *source of animal heat.*" That is, that the animal organism is a furnace, wherein the respiratory process furnishes the oxygen, and the elements of the food the fuel, the union of which produces the heat that keeps the animal machinery in motion. Animal diet consists of three different classes of food: the saccharine, the oleaginous, and the albuminous. The saccharine contains those farinaceous matters, gum, starch, dextrine, cellulose, that are convertible into sugar; the oleaginous includes all oily and fatty matters; and the albuminous those vegetable and animal substances that are directly convertible into the organic tissues. The saccharine are compounds of hydrogen and carbon, with a definite proportion of oxygen, just about enough to form carbonic acid and water. Now, in the oleaginous substances the proportion of carbon and hydrogen relatively to oxygen is far greater than we find in sccharine matters; hydrogen being highly combustible, a large amount of heat is generated in the process of their assimilation into the fatty and nervous tissues. The albuminous substances being so abundantly supplied with those nitrogenous elements that enter directly into the structure of nearly all the animal tissues, and the process of their assimilation is so direct and complete, there is comparatively very little evolution of heat.

It is evident from the foregoing discussion that of all articles of nutrition, oily and fatty matters are most valuable as heat-producing agents. The process of the digestion of these substances commences in the stomach, but is completed in the duodenum and the alimentary tract, and prepared for absorption into the circulation. As the burning of these elements—carbon and hydrogen—is dependent mainly upon the liberal supply of oxygen through the lungs, the number of respirations must be greatly increased to meet the demand upon that organ. But what is the fact? The number of respirations when the external temperature is high, while the body is engaged in moderate or even severe exercise, is less frequent than in cold weather. Again, in summer the amount of oxygen taken into the lungs at each inspiration is much less than in cold weather, on account of there being much less watery vapor inhaled under a low temperature. In the same volume of inspired air, therefore, in winter, there is a much larger proportion of oxygen. If the lungs fail to supply a due proportion of material for burning off an excess of hydrogen and carbon in the blood, the skin, liver, and kidneys, as co-laborers in the work of depuration, are called upon. An imposition upon them of an undue amount of work, must result in a retention of more or less effete matter in the blood, and consequent functional derangement of these excreting organs. The Esquimaux Indian will consume with much relish an incredible amount of train oil and tallow candles, and without other assistance will endure an intense degree of cold. The excessive demand upon his blood for these heat-producing agents enables him to enjoy them with the utmost gusto, and digest them with the greatest facility. The dweller within the tropics feasts upon the abundant production of fruits and vegetables with which nature so lavishly supplies him.

The enervating influence of a high atmospheric temperature incapacitates him for excessive physical labor; hence the beneficent Creator furnishes at his very door those articles of nutrition best suited to his condition. And here I may remark that the immense quantity of fat bacon consumed in our section of country in the summer, by the sedentary as well as the laboring population, predisposes the system to the development of malarial and biliary disorders. In protracted hot weather, an apology is offered for the use of alcoholic stimulants upon the plea that the loss of appetite requires the stomach to be worked up to a proper discharge of its duties. This, to some degree, is a delusive fancy; for, when there is not actual functional derangement of some organ, nature is the best monitor. The hard-working laborer burns off the excess of carbonaceous matter in his blood by the increased functional activity of his digestive organs, and he rarely complains of a disrelish for his meals. It is the man of sedentary habits who is the subject of tardy digestion. Nature usually indicates the amount of food required to supply the waste of the tissues. A state of physical inactivity is attended with a comparatively small degree of disintegration of muscular and nervous tissue; hence the demand for highly nutritious articles of diet is proportionally lessened. If, therefore, by alcoholic potations the stomach is stimulated to receive more food than it can comfortably digest and convert into the animal tissues, the excess either remains in the stomach or digestive canal for putrid fermentation, the fruitful source of colic and other ailments, or, if digested, it is thrown upon the organs of excretion for their elimination. Hence the habitual toper is so often the victim of flatulent distension of the stomach, acid eructions, and bilious diarrhœa.

The foregoing beautiful theory was promulgated by Leibig more than thirty years ago, and though in the main correct, yet not entirely so. He maintained that the office of the hydrocarbons is *solely* that of the production of animal heat, and that the albuminoids were exclusively employed in tissue-formation. Subsequent investigations, however, clearly indicate that the amyloids are not only to some extent tissue-formers, but they aid the albuminoids in their conversion into tissue. According to Leibig's theory, alcohol is incapable of being assimilated into the organic tissues, its atomic constitution being $C^4 H^6 O_2$. Although destitute of nitrogen, it may indirectly act as a nutrient, upon the principle of *conservation of energy*, in retarding the retrograde metamorphosis of the tissues. The demand for food is in proportion to the amount of disintegration and waste of tissue, and what is gained by retarding this process is so much added to the general fund of alimentary food.

Moleschett says: "He who has little can give but little, if he wish to retain as much as one who is prodigal of his wealth. Alcohol is the *savings-bank of the tissues*. He who eats little, and drinks alcohol in moderation, retains as much in his blood and tissues as he who eats more, and drinks no alcohol."

Again, may not alcohol act as a nutrient by supplying the material for chemical force whereby nitrogenous elements are converted into tissue? We know that upon the principle of the correlation of the forces, chemical force in the presence of the plasma of the blood, passes into vital force, whereby the assimilation of nutritive elements is accomplished. In wasting diseases, and voluntary and involuntary starvation, all the life-force is derived from the decomposition of the tissues. Alcohol may therefore do good work by furnishing the materials whereby urea is reconverted into tissue. The conservative energy of alcohol in asthenic diseases, by its almost entire absence from the breath and the secretions, even when given in large quantities. Dr. Anstie declares, "that in such instances its exciting and intoxicating powers appear to be in abeyance, and that the recovery from acute diseases, where this medicine has been successfully employed, is invariably more rapid and complete than it is in altogether similar cases which have been treated without alcohol."

Drs. Anstie, Thudielum, and Dupre have satisfactorily proved that a very small portion of the alcohol which is taken in the body is expelled as alcohol, and that therefore it must be employed in some way conservative to the general welfare of the animal. Dr. Anstie gave a dog weighing ten pounds "the liberal dose of 2000 grains of alcohol in ten days, and on the last day of the ten he administered 95 grains of the spirit as a final dose, and then two hours after killed the dog and immediately subjected the whole body—blood, secretion, flesh, membranes, brain, and bones—to rigorous analysis, and he found in the whole texture of the body only about 23½ grains of spirit. The other 1976 grains had clearly, therefore, been turned into something else within the living system."

We are well aware of the large quantities of brandy or whisky consumed by patients in typhoid fever and other diseases of low type, prolonged over days and weeks, affording meat, drink, and medicine, and without any apparent indications of intoxication, or unusual cerebral excitement. Its curative agency in wounds received from the bites of poisonous reptiles is universally acknowledged, being fairly attributable to its supply of vital force, sustaining the system until it is enabled to eliminate the poison through natural outlets.

In all low states of vital energy, from whatever cause, there is no agent from which a reaction is so rapidly secured. Dr. Anstie relates the case of an old soldier in the Westminster Hospital who had lived for twenty years upon a daily consumption of three-quarters of a pint of gin and a small piece of toasted bread, and who for this long period had maintained the structures of his body upon this simple diet. Dr. D'Lalor tells us that in a malarious country he lived upon wine and brandy, with very little solid food, for two years; and at the end of that time was not materially starved or emaciated. He also declares that he knows many vigorous and healthy men in London, "not only waiters, potmen, publicans, and the like, but tradesmen and merchants, who eat but little solid food, but have plenty of wine, gin, porter, etc."

Leibig stated that in temperance families, where beer was withheld and money given in compensation, it was soon found that the beer was twice paid for—once in money and a second time in bread. He also reported the experience of the landlord at the Peace Congress, the members of which were mostly teetotallers. He had to replenish the dishes of farinaceous food, pudding, pies, and confections, so frequently that he was astonished. It was found that the men made up the deficiency in wine with these articles of diet. This hint would be worth noting by those who are addicted to the inordinate use of alcoholic liquors. The liberal use of confectionery seems to keep in abeyance, to some degree, the inordinate desire for stimulants.

In further confirmation of the food property of alcohol, Dr. Anstie adduces the case of a young man, eighteen years of age, who was so reduced by a protracted attack of rheumatism that he was unable to retain anything upon his stomach. He lived for several days upon an allowance of twelve ounces of water and twelve ounces of gin per day. His recovery was very rapid and complete under this treatment, and though of very sober and temperate habits previous to this attack, yet he never indicated the slightest indications of intoxication or abnormal mental excitement. His pulse previous to the commencement of this treatment, was abnormally rapid and feeble, but it soon became more steady and full, until it returned to the natural standard of health.

"Dr. D'Lalor, before quoted, also mentions the case of a child only fourteen months old, suffering from inflammation of the lungs, and whose stomach could retain nothing but port wine. For twelve days it subsisted entirely upon wine; it was rapidly cured, with no wasting of any account; nor, although it drank large quantities of alcohol, was it ever intoxicated."

Those who live in malarious districts of country, and who have been compelled to fortify themselves against the debilitating effects of malarial poison by the very moderate use of bitter tinctures, either of bark of quinine dissolved in gin or whisky, will testify to its prophylactic agency, and the feeling of ennui and general malaise which ensues upon discontinuing the use of these preparations. It is also equally notable that a change in summer to a cool, healthy, bracing climate, in a few days releases one from not only dependence upon, but any desire for any artificial tonics or stimulants. Where prophylactics of the kind above mentioned have a salutary effect, the appetite and digestion are improved, attended with renewed physical vigor and nervous energy. It would appear, therefore, that when alcoholic drinks are required by the system, they create a desire and necessity for more food; and *vice versa*.

Steinmetz says: "I feel compelled to believe that, in advance of Leibig, *alcohol is absolutely generated in the digestive process of all animals*. It is well known that all the vegetables we eat contain starch; all the fruits contain sugar. Now, starch can be easily converted into sugar; the process of malting is a familiar instance. * * * The natural heat of the body is precisely adapted, in the healthy state, to effect a fermentation after having changed the starch into sugar, which last is constantly found in the blood. That alcohol has not been detected in the blood seems to result simply from the fact that it must be sought in arterial blood, or blood which has not lost a portion of its carbon *in transitu* through the lungs in the respiratory process." If this be the case, it would appear that in the healthy body there would be no necessity for alcohol, especially in a healthy climate free from malarial poison. We believe that general experience confirms this suggestion.

The belief is almost universal that the process of baking bread expels all the alcohol generated in the process of the fermentation of the gluten and starch of wheat flour; but Mr. Thomas Bolas, of London, informs us that when two ounces of alcohol are mixed with water and distilled, the distillate shows an appreciable quantity of alcohol. From his experiments with several samples of bread, he arrives at the conclusion that when a man has eaten one hundred pounds of bread he has consumed five ounces of pure alcohol.

Mr. John C. Lucas, L.R.C.S.E., in the *London Med. Times and Gazette*, recommends the internal use of alcohol in the anhydrous form, as rectified spirit, which contains about 85 per cent. of pure alcohol. Whisky, brandy, and gin should contain from 50 to 54 per cent. He objects to the latter on account of their frequent adulteration with noxious ingredients. When given internally, alcohol should be freely diluted with water, and disguised by some pungent aromatic, not only to conceal its disagreeable taste, but to annul any prejudices that the patient might have to its use. He sums up the advantages of alcohol: 1st, on account of the facility we have of regulating its quantity; 2d, when it is dispensed and labeled in the ordinary way as medicine, the friends and nurses of the patient will be more likely to give it at regular and specified intervals; 3d, the patient takes medicine more readily than food; and 4th, the nurse will not be so apt to take a portion for his own stomach's sake and his temporary necessities. In Mr. Lucas' experience with the use of alcohol, the stimulating effect he has found "more rapid and more lasting than when an equivalent of brandy (good) was used."

The use of fermented liquors is coeval with the earliest ages. The ancient preacher of righteousness (Noah) at one time fell a victim to its seductive allurements. The Apostle to the Gentiles counselled his son Timothy to "take a *little* wine for thy stomach's sake and thine often infirmities." All nations and all tribes of men find a temporary solace for care and trouble in the use of some form of fermented or distilled liquors. This fact would seem to indicate its necessity as an inherent want of the animal economy; but unfortunately so few, comparatively, heed the injunction of the Apostle to take a *little* for thine often *infirmities.* It is computed that alcohol destroys annually, in its own way, 10,000 persons in Russia, 50,000 in Great Britain, and 49,000 in this country. It is a double-edged sword that cuts for evil as well as for good. In the hands of the wise and prudent it is a powerful weapon of offence, gleaming with the lustre of victory over disease and death; in the hands of the thoughtless and simple, it cleaves the hopes and fortunes of many a crushed and bleeding heart. We well remember a clergymen, whose memory is now fragrant with the perfume of noble deeds and the kindliest sympathies, whose prejudices were so inveterate against alcohol, that he permitted a favorite horse to die before compromising his convictions by saving the life of his animal with a bottle of whisky. On the other hand, a sober and temperate youth, brought up in the strictest household of the prophets, was advised to use alcohol for the relief of a serious pulmonary disease, but all the convictions of his early training impelled him, at first, not to heed the physician's prescription. He finally yielded, however, and was rewarded with freedom from disease, but cursed with a lifetime subjugation to a relentless tyrant.

Let us, therefore, as physicians, diagnosticate disease with carefulness, precision and certainty; cautiously and wisely discriminate the weaknesses and proclivities of our patients, and prescribe this potent remedy, ever mindful of its deadly fascinations, as well as its life-giving energy.

